# The gene-diet associations in postmenopausal women with newly diagnosed dyslipidemia

**DOI:** 10.1007/s12603-017-0877-4

**Published:** 2017-02-08

**Authors:** Bogna Grygiel-Górniak, E. Kaczmarek, M. Mosor, J. Przysławski, J. Nowak

**Affiliations:** 1 Department of Rheumatology and Internal Diseases, Poznan University of Medical Sciences, Poznan, Poland; 2 Department of Bromatology and Human Nutrition, Poznan University of Medical Sciences, Poznan, Poland; 3 Department of Bioinformatics and Computational Biology, Poznan University of Medical Sciences, Poznan, Poland; 4 Department of Molecular Pathology, Institute of Human Genetics, Polish Academy of Sciences, Poznan, Poland

**Keywords:** Polymorphisms of PPARγ2 and ADRβ3 genes, dietary habits, postmenopausal dyslipidemia

## Abstract

**Objectives:**

The aim of this study was to determine the relationship between polymorphisms of peroxisome proliferator activated receptor - PPAR gamma-2 (Pro12Ala, C1431T) and beta 3-adrenergic receptor - ADRB3 (Trp64Arg) and dietary habits in a group of postmenopausal women who were not under hypolipidemic treatment.

**Design:**

Genetic, nutritional and anthropometric parameters were measured in 213 dyslipidemic (LDL ≥115 mg/dL) and 58 normolipidemic (LDL<115) postmenopausal women. The PCR-RFLP method were used to determine the distributions of selected alleles and genotype frequencies. Dietary intake of basic components and fatty acids was obtained from a 7-day weighed food record and the bio-impedance method was used to determine nutritional status.

**Results:**

Nearly 79% of analyzed women were in the firsttime-diagnosed dyslipidemic state. The dyslipidemic subjects were characterized with higher intake of energy, fat, and saturated fatty acids (SFA). The analysis of the same polymorphisms showed association at the P value <0.05 with nutrients (fat, SFA, and polyunsaturated fatty acid - PUFA and saccharose) and elevated LDL level. Higher PUFA intake in a group of women with the protective Ala12/X polymorphism did not increase the risk of dyslipidemia even though they were characterized by visceral distribution of fat. The Arg64/X polymorphism and higher intake of energy, fat, and arachidic acid intake (C20:0) were associated with dyslipidemic state.

**Conclusion:**

Both nutritional and genetic factors are related to lipid profile. The identification of gene-diet associations is likely to provide useful information about the etiology of postmenopausal dyslipidemia and help in effective treatment.

## Introduction

Many European populations, including Poland, represent countries of high risk of cardiovascular diseases (CVD) (1). Recent studies of Polish population – NATPOL 2011 showed that nearly 61% of adult Polish inhabitants (about 18 million) have dyslipidemia (2), which is the main risk factor of CVD (3). The European Study on Cardiovascular Risk Prevention and Management in Usual Daily Practice showed that general prevalence of dyslipidaemia in Europe is about 57.7% of patients with at least one major risk factor for CVD (4) and ranges between 29% in Switzerland (5) till 59.1% in Italy (6) and 65.4% in Spain (7). Many genetic factors (including PPARγ2 or ADRβ3 genes) show variable association with dyslipidemia (8, 9) and can be modulated by selective nutrients intake (10, 11). Because polyunsaturated fatty acids and prostanoids (12) are the major natural ligands of the PPARγ2 gene, the quality of fat may affect transduction of metabolic signals (13). In animal studies an increase in PPARγ mRNA in adipose tissue was observed after a high-fat diet supplement (8). Moreover, C1431T polymorphism of PPARγ2 gene is associated with metabolic disorders, dyslipidemia, and CVD development (14-16). Besides PPARγ2 gene, ADRβ3 gene, located mainly in adipose tissue, also is involved in the regulation of lipolysis and thermogenesis (17); and the presence of Arg64 allele is related to the increased chances of gaining weight in Caucasians (25 year observation of obese subjects) (18).

Some studies have reported on the combined role of genetic (e.g., Pro12Ala genotype) and lifestyle (such as dietary fatty acid ratio) factors in metabolic disorders (insulin resistance or BMI value) (10, 11). However, usually, in patients with metabolic disorders the already used medications could affect glucose and lipid concentrations or the expression of the PPARγ2 gene (e.g., thiazolidinediones and statins). Taking into consideration the above mentioned data, we attempted in our study to estimate the relationship between specific polymorphisms, nutritional status and dietary habits in a group of postmenopausal women, who were never diagnosed of or treated for dyslipidemia and show, which of the genetic factors may play an important role in the treatment strategies in this group. Moreover, we would like to indicate, which of the nutritional habits should be changed to treat or prevent the exacerbation of dyslipidemia. 

## Material and methods

### Analyzed group

In this study, 1,423 women, aged 49 to 75 years, were recruited and they underwent standard health checkups at a metabolic outpatient clinic. From this group, we selected postmenopausal women who did not undergo any hypolipidemic or hypoglycemic treatment. After the gynecological interview (minimum 12 months of amenorrhea or bilateral oophorectomy), the hormonal assessment based on the measurement of follicle-stimulating hormone (FSH) confirmed the postmenopausal period. Women earlier diagnosed with or treated for dyslipidemia, severe cardiovascular diseases, endocrinological disorders, renal or liver dysfunction, cancer or the ones supplemented with minerals/vitamins/fitosterols were excluded from the study. Finally, we selected 271 postmenopausal women, who undergo biochemical, nutritional, and genetic evaluation. The selection was random because PPARγ2 and ADRβ3 polymorphisms were unknown at the time of recruitment. The ethical approval was obtained from the respective local Bioethical Commission of Poznan Medical University, Poland, nr 792/09, and the guidelines proposed by the Declaration of Helsinki were followed. Written informed consent was obtained from all subjects before the commencement of the study. 

### Anthropometric measurements

Anthropometric measurements were directly taken in accordance with the International Standards for Anthropometric Assessment (19) by trained interviewers. Women’s weight, height, waist circumference, blood pressure, and bioimpedance were measured in the morning, using a standardized procedure. Body mass was determined in underwear in the standing upright position with electronic scales with a precision of 100 g (SECA scale). The height was determined to the nearest 0.1 cm. Waist circumference was measured at the midpoint between the lower margin of the least palpable rib and the top of the iliac crest, using a flexible measure to the nearest 0.1 cm. Body mass index was calculated as weight/height squared (kg/m2) and waist-to-hip ratio (WHR) as the proportion of waist-to-hip circumferences (20). A bioimpedance analyzer with a single frequency of 50 kHz (Bodystat 1500, Bodystat Ltd., United Kingdom) was used to assess the fat content to the proportion of total body mass. 

### Biochemical evaluation

After 12 hour fasting, venous blood samples were collected from all patients at 7 A.M. Serum samples were taken from clotted (15 min, room temperature) and centrifuged blood (15 min, 3 000 × g). Serum was separated and directly used for the assay. The obtained samples were used for the measurements of FSH levels and plasma lipid profile. The concentration of FSH was measured to confirm the postmenopausal age by chemiluminescence assays (Roche Diagnostics). The lipid profile was assessed with enzymatic colorimetric assays (Cobas Integra 400 Plus; Roche Diagnostics, Mannheim, Germany). Low density lipoprotein (LDL) cholesterol was calculated using Friedewald’s formula (21).

According to the recommendation of European Society of Cardiology (ESC) and the European Atherosclerosis Society (EAS) the characteristic SCORE (Systematic Coronary Risk Estimation) was calculated for whole group of women (n=271). Estimated SCORE (based on data for no-smoking women aged 60) was within the range of 1%–5%. The assessed coronary risk was moderate and thus LDL-cholesterol of 3 mmol/L (less than 115 mg/dL) should be considered as a target value in the analyzed group (2). The value of LDL <115 is also recommended by ESC as target in general population (without diagnosed CVD or diabetes mellitus, similar to the group in this study) (2, 22). Considering this evidence every subject with LDL level ≥115 mg was classified as dyslipidemic, while with LDL <115 mg/dL as normolipidemic. The lipid accumulation product (LAP) was calculated using the formula: LAP = (WC – 58) × TG (nmol/L) (23, 24). 

### Dietary evaluation

The food intake was assessed at a 24 hour interval for 7 days and all women were on normal diet (traditional Polish diet). The estimated daily food rations (DFR) enabled the analysis of energy intake, basic nutritional components consumption (protein, fat and carbohydrates) and selected fatty acids intake (including SFA, MUFA, PUFA). The results of the questionnaire were analyzed with both the quantitative and qualitative analyzes of the subjects’ daily diets using computer databases for Microsoft Access 2000. The food intake recommendations of the National Institute of Food and Nutrition in Warsaw, Poland, were taken in to account to determine whether the Recommended Dietary Allowances (RDAs) specific for age, sex, ideal body mass, height, and physical activity were fulfilled (25). Dietary cholesterol was compared with the nutritional prophylaxis recommendations at the level of 200 mg (recommended in dyslipidemia) (20, 25). 

### Genotyping

Genomic DNA was isolated from venous blood samples according to the manufacturer’s protocol (Gentra Puregene Blood Kit; QIAGEN, Venlo, Limburg). Genotypes of the Pro12Ala (rs1801282) and Trp64Arg polymorphism (rs4994) were determined by applying a TaqMan genotyping assay (Life Technologies, Carlsbad, CA). As a quality-control measure, negative controls and approximately 5% of samples were genotyped in duplicate to check genotyping accuracy. The controls for each of the genotypes of both single nucleotide polymorphisms were run in parallel. An allelic discrimination assay was performed on an ABI7900HT or on CFX96 Touch Real-Time PCR Detection System (Bio-Rad Laboratories, Inc, Hercules, CA). C1431T (rs3856806) genotyping was performed using PCR-restriction fragment length polymorphism (PCRRFLP) analysis. Eco72I cleaves the PCR product from wildtype DNA to generate fragments of 127bp and 43bp, but does not cut products containing the variant allele. PCR-digests were analyzed on 2.5% agarose gels. The determination of the ADRβ3 variant was performed using the PCR method as described by Sivenius and colleagues (26). The genotypes were determined as Trp64Trp, Trp64Arg, and Arg64Arg without earlier knowledge of the patients’ status. 

### Linkage disequilibrium block determination and haplotype construction

The genotype data were used to construct the haplotypes between the two polymorphisms by using Haploview 4.2 software (Broad Institute, Cambridge, MA) to evaluate linkage disequilibrium (LD). Linkage disequilibrium between the single nucleotide polymorphisms used in haplotype analysis was measured using a pairwise D’ statistic. The structure of the LD block was examined with the method proposed by Gabriel and colleagues (27) using the 80% confidence bounds of D’. The haplotype frequencies were calculated based on the maximum likelihood method with Haploview 4.2 software. Finally, the associations between haplotypes and obesity status were checked. Specific haplotype frequencies were compared among lean and obese women (chi-square test). 

### Statistical Analysis

The Shapiro-Wilk test was used to determine if the continuous variables were normally distributed. Since the results were not consistent with normal distribution, nonparametric methods were used for the statistical analysis. Continuous data were shown as mean ± standard deviation. The hypothesis that the differences between analyzed anthropometric and metabolic factors in the analyzed groups were significant was tested by Mann-Whitney U-test. As the number of Ala12Ala homozygotes were small (one lean woman and nine obese women), compared to Pro12Pro homozygotes, they were collapsed with Pro12Ala heterozygotes for all the analyzes. Similarly, C1431T heterozygotes and T1431T homozygotes were collapsed together, as well as Trp54Arg heterozygotes and Arg64Arg homozygotes. Allele frequencies were estimated using the gene-counting method, and an exact test was performed to identify departures from Hardy-Weinberg proportions (Court lab–HW calculator.xls). Genotype and allele frequencies according to normolipidemic (LDL <130 mg/ dL) and dyslipidemic state (LDL ≥130 mg/dL) were tested by Mann-Whitney U test. The association of analyzed genotypes with anthropometric and nutritional parameters in normo- and dyslipidemic women was also tested by Mann-Whitney U test. The odds ratio (OR) with a 95% confidence interval (CI) for gene polymorphisms according to normolipidemic (LDL <115 mg/dL) and hyperlipidemic state were determined. A P value <0.05 was regarded as statistically significant. The statistical analyzes were performed with STATISTICA 12 (including STATISTICA Medical Package 2.0; StatSoft, Inc. 2014 software) and SPSS 22 (IBM, Inc, Chicago, IL, USA).

**Table 1:**
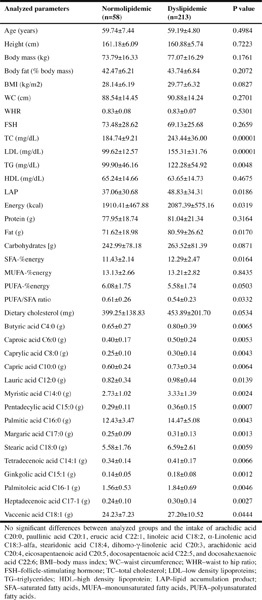
Anthropometric and nutritional characteristics of postmenopausal women

**Table 2:**
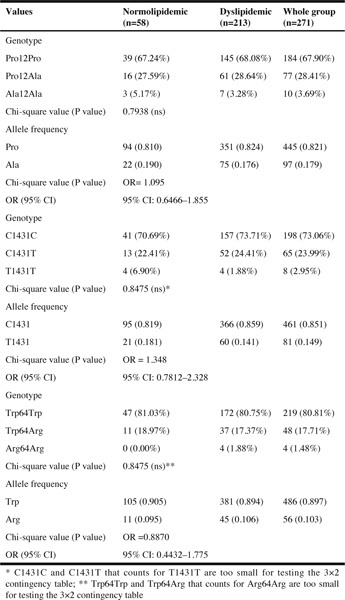
Genotype and allele frequencies of the Pro12Ala and C1431/X PPARγ2 and Trp 64Arg of β-adrenergic receptor gene polymorphisms according to normolipidemic (LDL <130 mg/dL) and dyslipidemic state (LDL ≥130 mg/dL). Data are n (%) for genotypes and n (frequency) for alleles

## Results

The anthropometric parameters of dyslipidemic and normolipidemic subjects were comparable in both groups (Table 1). All women were overweight (BMI >25 kg/m2) and characterized with visceral distribution of fat (WC >80 cm) (28). Besides differences in LDL level (used as independent variable to classify the patients as normolipidemic or dyslipidemic) we also observed the differences in TC, TG, and non-HDL cholesterol between analyzed groups. The lipid accumulation product (LAP) was significantly higher in dyslipidemic group. The intake of energy as total fat and as saturated fatty acids (SFA) expressed in percent of total energy intake and analyzed individually (as butyric, caproic, caprylic, capric, lauric, myristic, pentadecylic, palmitic, margaric, and stearic acid, C4:0 – C20:0) and as monounsaturated fatty acids (such as tetradecanoic, ginkgolic, palmitoleic, heptadecanoic, and vaccenic acid, C14:1 – C18:1) were higher in dyslipidemic women. There were no statistically significant differences between the intake of total amount of PUFA (expressed as percent of energy intake) and selected PUFA in analyzed group (linoleic, α-Linolenic, stearidonic, dihomo-γ-linolenic, arachidonic, eicosapentaenoic, docosapentaenoic, and docosahexaenoic acid). The dietary cholesterol intake was higher in dyslipidemic group, while the ratio of PUFA to SFA was lower. 

## Discussion

Several factors were proposed to be associated with dyslipidemia including dietary habits, physical activity, and genetic background (9, 29-32). However, the studies describing such relations in postmenopausal age are very scarce (33, 34), and to our knowledge neither of them describe the diet–gene interaction in newly diagnosed postmenopausal dyslipidemia. In this study, we found both nutritional and genetic associations with the dyslipidemic state.

The analyzed groups of women (Table 1) were overweight (BMI ≥25<30 kg/m2) (20, 28). The waist circumference (WC) and WHR ratio over-crossed recommended values in normolipidemic and dyslipidemic subjects (WC >80 cm and WHR >0.8) (28). These parameters show the central deposition of fat, which is considered not only as a risk factor of CVD diseases in postmenopausal age (35), but also as a risk factor of breast cancer (36). Beside this, LAP (a novel index of central lipid accumulation) was found to be elevated in both groups and was significantly higher in dyslipidemic women. In European population, the optimal cut-off value of LAP for the screening of metabolic syndrome is 33.28 in females (37). Increased LAP in both groups shows not only a central lipid accumulation, but also predicts the risk of metabolic syndrome (23, 38) and CVD development (23, 37).

**Table 3:**
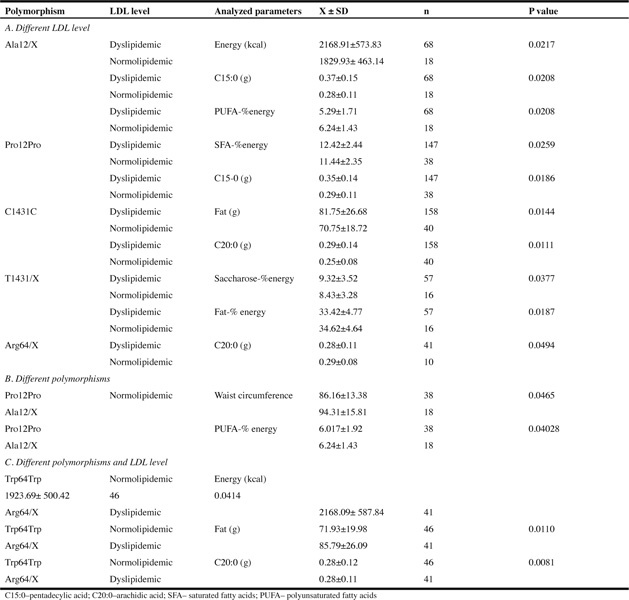
The association of analyzed genotypes with anthropometric and nutritional parameters in normo- and dyslipidemic women

The intake of energy, as fat and saturated fatty acids were higher in dyslipidemic women, while PUFA/SFA ratio was lower (Table 1). Mainly the excess of SFA causes an elevated LDL level and the substitution of these acids for polyunsaturated fat in the diet results in the decrease of both total cholesterol and LDL concentration (39, 40). Beside the excess of SFA, low amount of PUFA were noted in both groups, what influences on high LDL level (41, 42). The proper amount of saturated acids range from 8%–9% of energy in DFR, MUFA 13%–14%, and PUFA at 8%–9% (Mediterranean diet) (37). MUFA were within the recommended range and may cause quite high HDL (“protective”) level in both normolipidemic and dyslipidemic group (37, 43, 44).

In this study, we did not confirm the differences between gene distribution and the presence of dyslipidemia (Table 2). However, we have observed some associations of nutritional and genetic background with the level of LDL. We have showed that Ala12 genotype may be related to the lower risk of dyslipidemia (Table 3, Part B) and this genotype together with the dietary factors (higher intake of PUFA) seems to play a protective role. Because, even though Ala carriers had higher waist circumference, they remained normolipidemic. Similar to our study, Robitaille at al., have showed that each 10 g increment in fat intake was associated with an increase of 1.2 cm in waist circumference among Pro12/Pro12 homozygotes, whereas no significant change in WC was observed among carriers of the Ala12 allele (11). The normolipidemic state in Ala12/X polymorphism can also be partially explained by the fact of higher intake of PUFA, which have beneficial influence on the lipid profile and decrease the risk of CVD development (30-33).

Dyslipidemic women with Arg64/X polymorphism of the ADRβ3 were characterized with higher intake of energy, total fat, and arachidic acid (C20:0). Thus, this polymorphism seems to be related to dyslipidemic state and can increase the lipid disorders if it is associated by higher intake of proatherogenic nutrients. This evidence is confirmed by the study of Asian (45) and Caucasian populations (18), which have shown that Arg64 allele is associated with obesity development. Moreover, obese subjects with this allele are predisposed to lipid disorders (higher TC, LDL, and TG) (46). Therefore, the association of the Arg64 allele with an inadequate amount and quality of fat in daily food rations of the analyzed women can be predisposed to postmenopausal dyslipidemia. We conclude that both genetic background and nutritional habits are related to health status in an aging population (18, 45, 46,47). 

## Conclusion

To our knowledge this is the first study, which showed a genetic associations of ADRβ3 and PPARγ2 variants and eating behavior in postmenopausal women with dyslipidemia who did not undergo hypolipidemic treatment. The dyslipidemia was first time diagnosed in nearly 80% of postmenopausal women, which suggest the need for early diagnosis, dietary modification, and/or hypolipidemic treatment in postmenopausal women to prevent CVD development. According to the recommendations of ESC and the European Atherosclerosis Society (EAS) women with LDL ≥115 mg/ dL should undertake lifestyle interventions including dietary modification and increased physical activity. Therefore, lower intake of fat and SFA and higher intake of PUFA should be recommended. Alongside, the presented results suggest that some polymorphisms together with the selected nutrients are related to dyslipidemia. Therefore, Ala12 (protective) and Arg64 allele (predisposing to dyslipidemia) may play an important role in the treatment strategies in long-term weight changes in viscerally overweight postmenopausal women with newly diagnosed dyslipidemia.

*Ethics declaration*: All experimental procedures were conducted in accordance with the guidelines in the Declaration of Helsinki and approved by the Bioethics Committee of Poznan University of Medical Sciences in Poland.

*Acknowledgments*: B.G.G designed the study and supervised the practical carrying out of the clinical trial; M.M. conduct genetic measurement; E.K. and B.G.G. do statistical analysis; J.P. and J.N. analyzed the data; B.G.G. wrote the manuscript and had the primary responsibility for final content. All authors read and approved the final manuscript.

*Funding source*: This study was supported by the Polish National Science Center (NSC) under grant No. N404 504 638.

*Conflict of Interest Disclosures*: The authors do not have any conflicts of interest
